# Migrating foreign bodies of penis: a case report and literature review

**DOI:** 10.1186/s12610-024-00224-3

**Published:** 2024-05-06

**Authors:** Bo Yang, Ying Ke, Aixin Qiu, Lijie Wen, Xiaolong Xv, Xiaoyun Liu, Yue Zhang

**Affiliations:** Department of Urology, The Second Hospital of Dalian Medical University, Dalian, 116021 China

**Keywords:** Foreign body, Retained penile needles, Urologic emergency, Case report, Literature review, Corps étranger, Rétention d’Aiguilles péniennes, Urgence urologique, Cas clinique, Revue de la Littérature

## Abstract

**Background:**

Only a few cases have been reported about active foreign body implantation in the cavernous body of the penis.

**Case presentation:**

A 47-year-old man inserted two needles from the glans penis into the bilateral penile sponge body. Subsequently, two needles migrated through the penile cavernous body into the pelvic cavity. Attempts to remove the needles through the penis were unsuccessful. Eventually, after a duration exceeding one month, the displaced needles were removed in stages from the buttocks.

**Conclusion:**

A few cases of intracavernosal-injection-therapy-associated needle breakage and retention have been reported globally. And this is the first case in China documenting the migration of foreign bodies within the penile region. In this condition, it is of utmost importance to engage the expertise of experienced andrologists to minimize the risk of excessive manipulation, thereby ensuring that inadvertent deep penetration of the needle into the penile tissue is prevented. In case the foreign body has migrated deeper into the tissues and the patient does not exhibit any specific symptoms or risks of macrovascular injury-related bleeding, close surveillance of its movement can be implemented. Surgical intervention can be initiated once the foreign body has reached a suitable position. Moreover, a psychiatric evaluation should be recommended for patient to discover any underlying mental health disorders.

## Résumé

**Contexte:**

Seuls quelques cas ont été rapportés concernant l’implantation active d’un corps étranger dans le corps caverneux du pénis.

**Présentation du cas:**

Un homme de 47 ans a inséré deux aiguilles, par le gland du pénis, dans les corps spongieux du pénis. Par la suite, les deux aiguilles ont migré à travers le corps caverneux du pénis jusque dans la cavité pelvienne. Les tentatives pour retirer les aiguilles à travers le pénis ont été infructueuses. Finalement, après une durée de plus d’un mois, les aiguilles déplacées ont été retirées, par étapes, au niveau des fesses.

**Conclusion:**

Quelques cas de rupture et de rétention d’aiguille associés au traitement par injection intracaverneuse ont été signalés dans le monde. Il s’agit ici du premier cas en Chine qui documente la migration de corps étrangers dans la région du pénis. Dans cette situation, il est de la plus haute importance de faire appel à l’expertise d’andrologues expérimentés pour minimiser le risque de manipulation excessive, garantissant ainsi que la pénétration profonde par inadvertance de l’aiguille dans le tissu pénien est prévenue. Dans le cas où le corps étranger a migré plus profondément dans les tissus et que le patient ne présente pas de symptômes spécifiques ou de risques de saignements liés à une lésion macrovasculaire, une surveillance étroite du mouvement du corps étranger peut être mise en œuvre. L’intervention chirurgicale peut être initiée une fois que le corps étranger a atteint une position appropriée. Enfin, une évaluation psychiatrique devrait être recommandée à la recherche de tout trouble sous-jacent de santé mentale.

## Introduction

Occasional reports have emerged regarding needle breakage within the penis during intracavernosal injection therapy (ICI) for patients afflicted with erectile dysfunction (ED). Nevertheless, there is a scarcity of documented cases regarding voluntary self-needle insertion into the penis and the ensuing displacement. In July 2023, our hospital encountered a unique case involving the self-insertion of two needles into the bilateral cavernous body.

## Case presentation

**Fig. 1:**
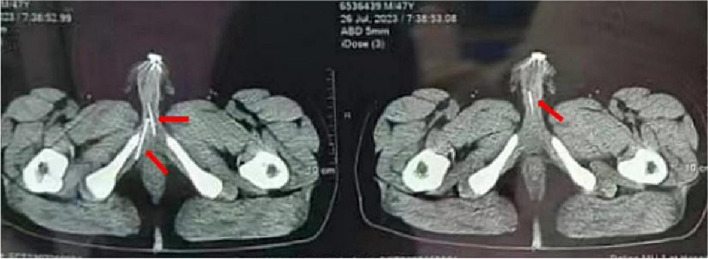
July 26, 2023, plain CT of the pelvis. The arrows show needles in the cavernous body of the penis on both sides

A 47-year-old married male, with a history of hypospadias and normal erectile function, independently inserted 10 steel balls of varying sizes subcutaneously through small incisions of the foreskin into the coronal sulcus, root of the penis, and the scrotum three months ago. Despite this behavior, the patient denied any previous history of mental disorders. Recently, the patient procured several elongated hollow needles via online sources, typically utilized for ICI, but he denied any usage for injection purposes. The needles had a length of 5 cm and a diameter of 0.5 mm. Approximately 18 h prior to admission, the patient experienced a nocturnal erection and self-inserted two needles along the long axis of the penis into the bilateral penile sponge body from the glans penis. He complained of mild pain during the insertion of the foreign objects, but did not experience any significant pain or discomfort thereafter. Urination remained normal without any presence of blood. However, due to an intolerable level of distress localized at the base of the penis, the patient made the decision to seek medical attention in the emergency room roughly 11 h subsequent to the needle insertion. He underwent a pelvic Computed Tomography (CT) examination, which revealed needle-shaped foreign bodies within the cavernous bodies bilaterally in the penis. In addition, a total of 10 nodular dense shadows were observed within the penis and scrotum (Fig. 1). The patient requested the extraction of the needles, yet declined surgical intervention due to apprehensions regarding potential postoperative ED. Following 7 h of hesitancy and careful consideration, the patient was admitted to the hospital on an emergency basis due to intensifying unbearable pain at the base of the penis and perineum.

**Fig. 2:**
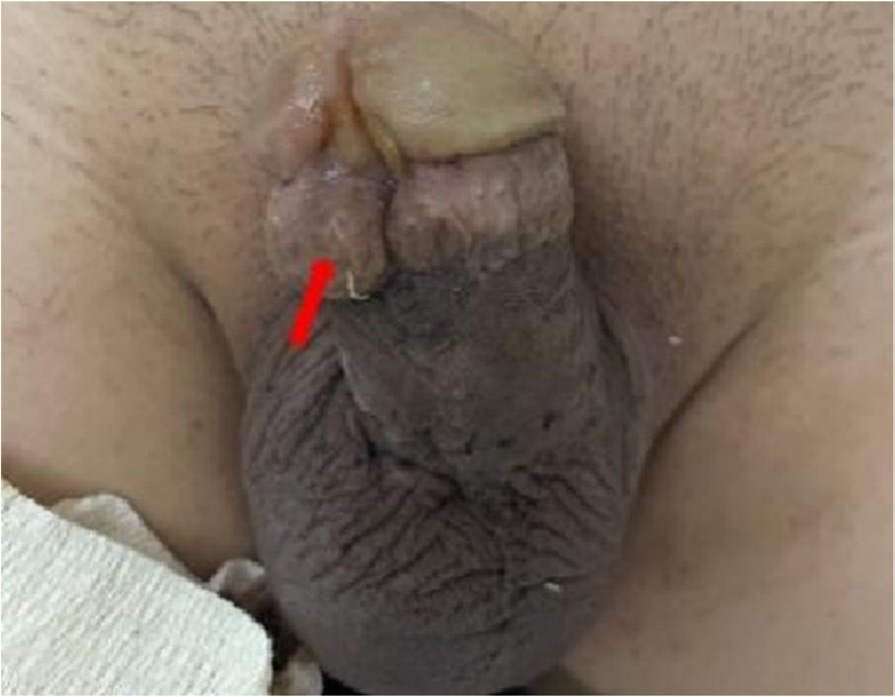
Appearance of the patient’s penis: glans cleft combined with anterior hypospadias, external urethral opening in the ventral coronal sulcus. The metal beads hidden beneath the foreskin are indicated by the arrow

Physical examination revealed pinhole-like marks on the surface of both sides of the glans, along with the combination of glans cleft and anterior hypospadias, and external urethral opening in the ventral coronal groove (Fig. 2). Numerous bead-like foreign bodies, approximately 5–10 mm in diameter, were perceptible within the subcutaneous tissues of the coronal groove, root of the penis, and scrotum. The needles could not be detected through palpation of the penis.

**Fig. 3:**
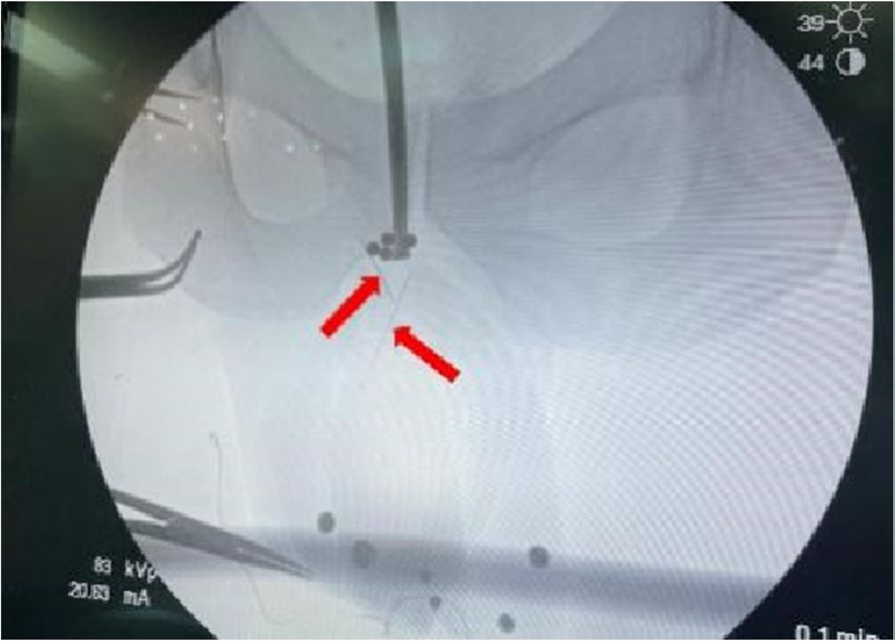
Intraoperative X-ray of the patient’s first surgery on July 26, 2023. The arrows show the needles

**Fig. 4:**
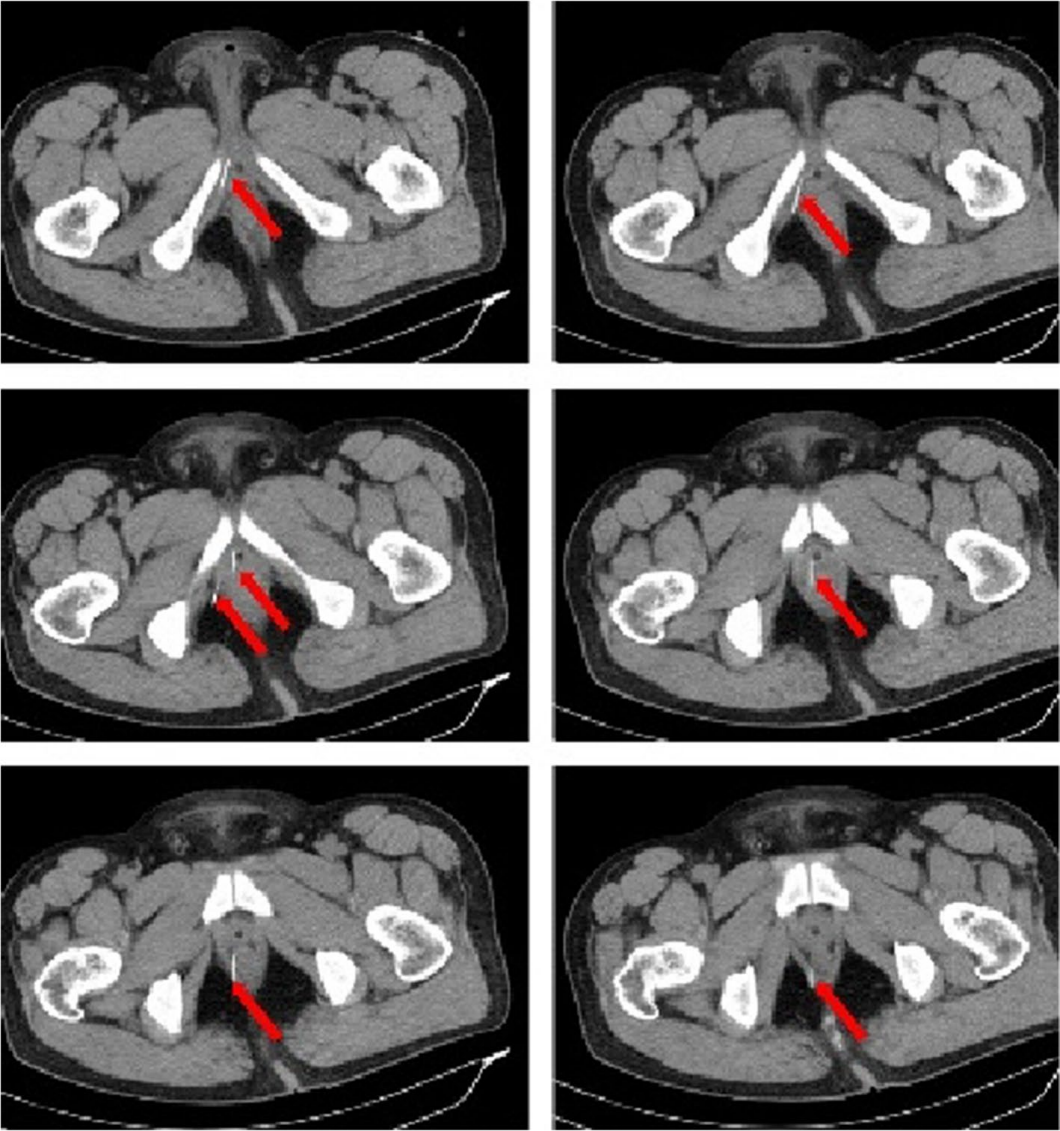
July 27, 2023, CT of the pelvis. The arrows point to the needles

**Fig. 5:**
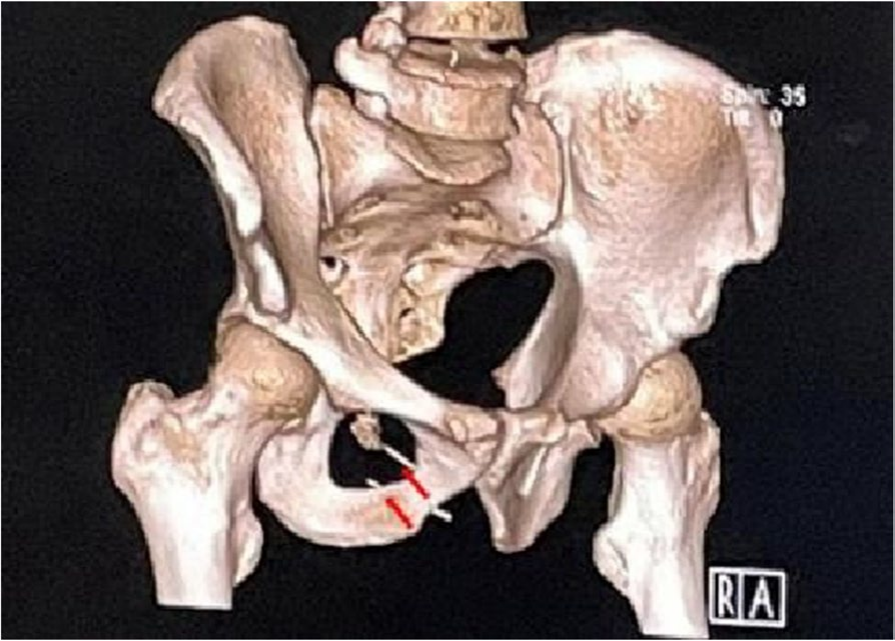
July 27, 2023, a 3D reconstruction of pelvis. The arrows point to the needles

**Fig. 6:**
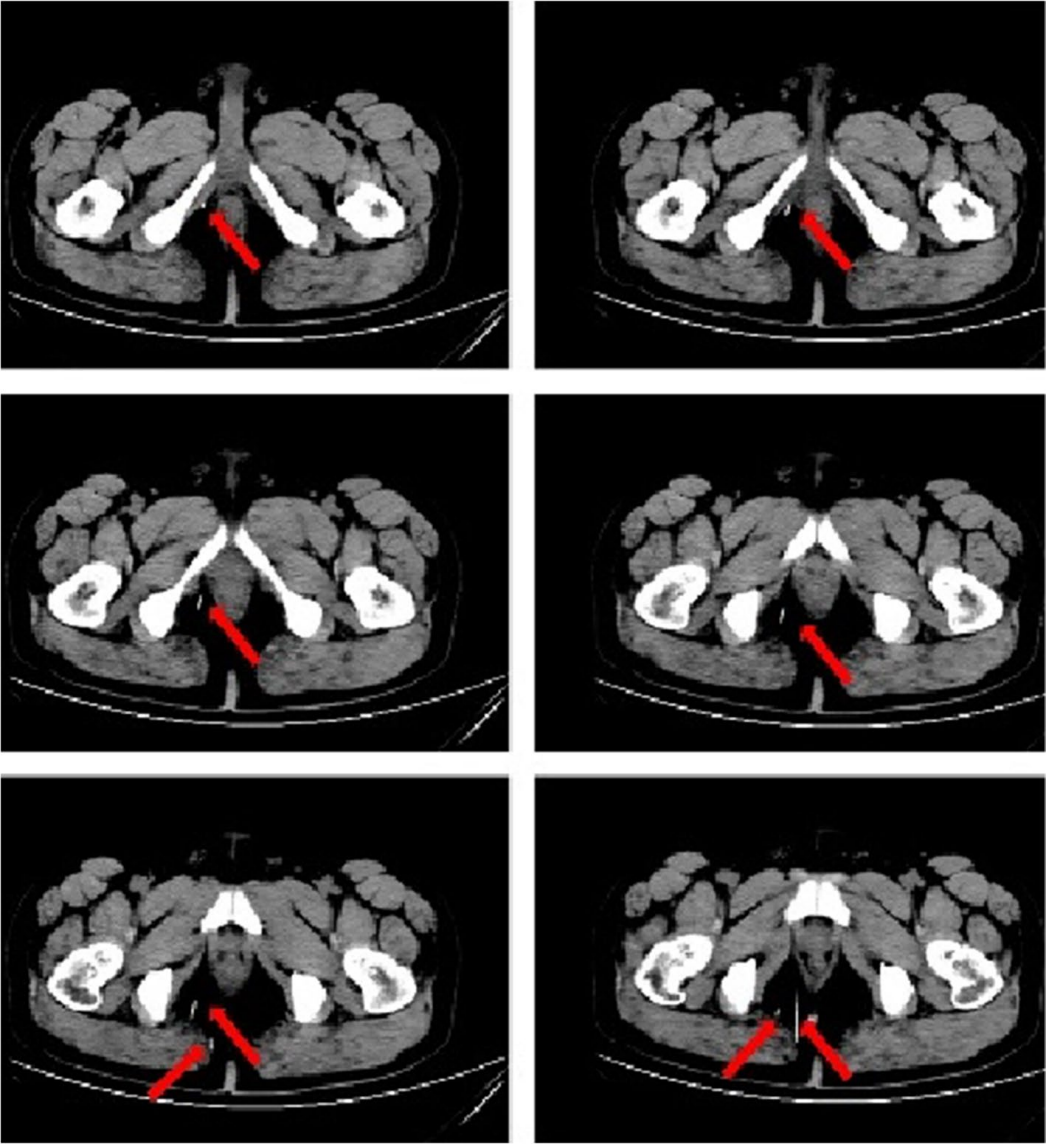
July 31, 2023, CT of the pelvis. The arrows point to the needles

**Fig. 7:**
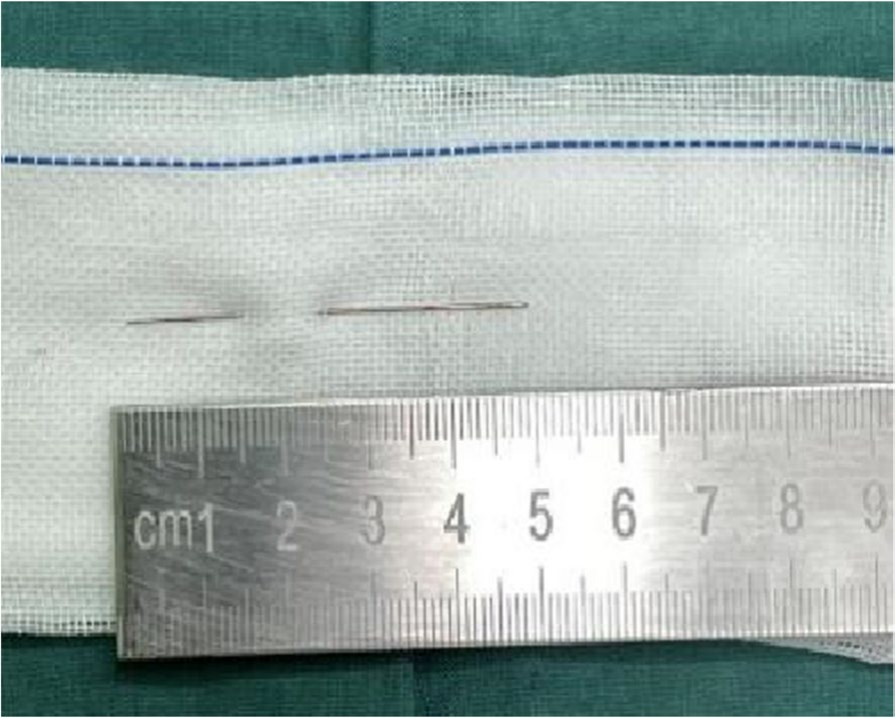
August 2, 2023, a needle extracted from the patient’s right buttock

**Fig. 8:**
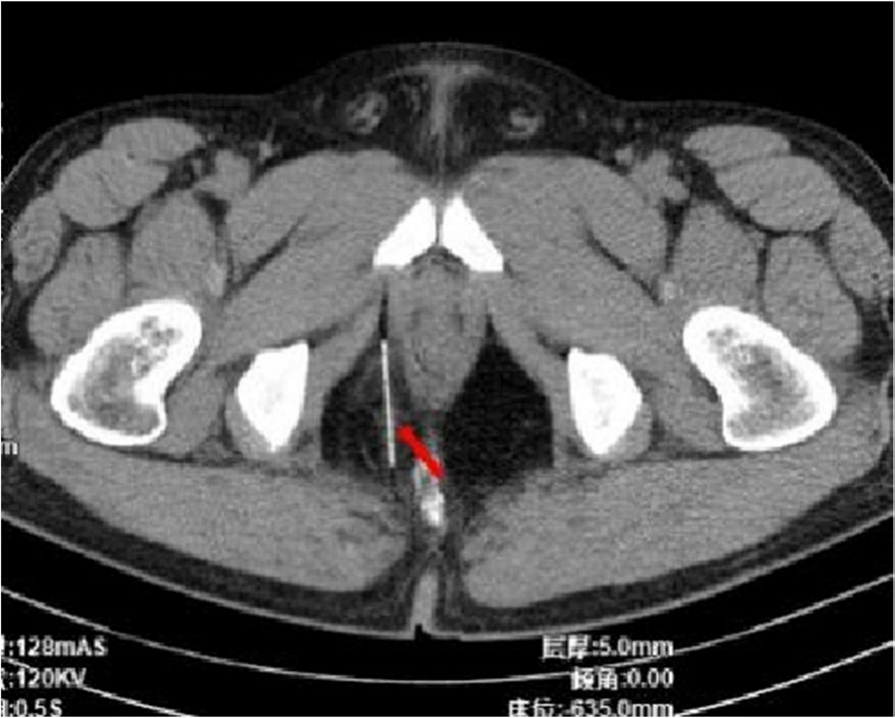
August 7, 2023, CT scan of the pelvis. The arrow points to the residual needle

Twenty two h following the incident, the patient underwent surgical exploration in urology department. Intraoperative X-ray guidance was employed to successfully locate the needles in the posterior section of the penile body, with their anterior ends extending beyond the pubic symphysis (Fig. 3). Under general anesthesia, a circular incision was made on the foreskin, 1 cm away from the base of the penile body’s surface. Through dissection of the superficial fascia towards its proximal end, the tunica albuginea of the corpus cavernosum was exposed up to the pubic symphysis. A longitudinal incision was made on the lateral side of the tunica albuginea of the right cavernosum; however, no foreign body was detected within the cavernous tissue of the penis. Following the verification of adequate hemostasis, the incision was meticulously closed in a layered manner. On the second postoperative day, a pelvic CT scan was conducted, revealing significant displacement of the needles into the deep tissues (Figs. 4 and 5). The needles pierced the cavernous body of the penis and reached the pelvic floor, thereby inducing a gradual amelioration of the patient’s distress. Instead of pursuing trans-perineal surgery, close surveillance was selected as the foreign body was primarily situated within the soft tissues of the pelvic floor, distanced from major blood vessels and the rectum. On the fifth day following the surgical procedure, a subsequent pelvic CT scan revealed further migration of the two needles into the sacrococcygeal region (Fig. 6). However, on the seventh postoperative day, the patient began experiencing deteriorating perineal pain and unbearable acupuncture-like sensation in the right buttock when seated. Physical examination revealed an elevation of the cutaneous surface in the right buttock proximal to the gluteal cleft, with noticeable tenderness and a palpable sensation of a foreign body beneath the skin. Therefore, an emergency procedure was performed under local anesthesia. Intraoperative X-ray revealed the presence of one needle within the deep soft tissue of the pelvic floor, while another needle was oriented perpendicular to the body’s surface in the right buttock. With intraoperative X-ray guidance, an incision of 1 cm in length was made on the skin, which was then probed downward for 2 cm, resulting in the complete extraction of a hollow needle with a length of 5 cm and a diameter of 0.5 mm (Fig. 7). Following the surgery, the localized pain symptoms were reduced. Pelvic CT examination on the 12th postoperative day confirmed the stability of the residual needle’s position. The patient was instructed to closely observe it and await an appropriate opportunity for the removal of the foreign body (Fig. 8).

**Fig. 9:**
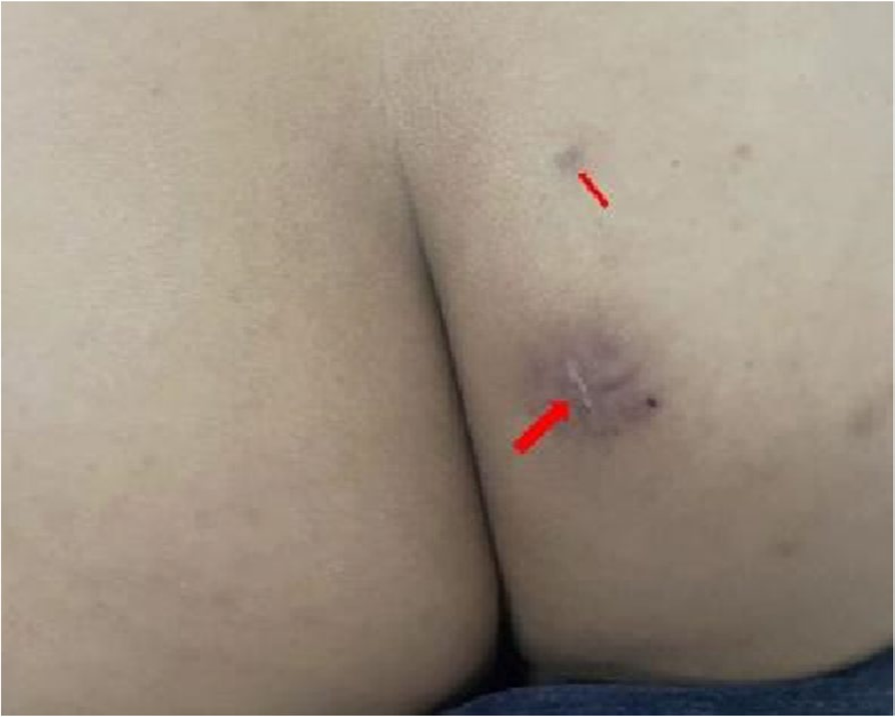
The departure positions of the two foreign bodies are indicated by the arrows. The thick arrow represents the first exit position and the thin arrow represents the second exit position. Both needles were removed from the right buttock

**Fig. 10:**
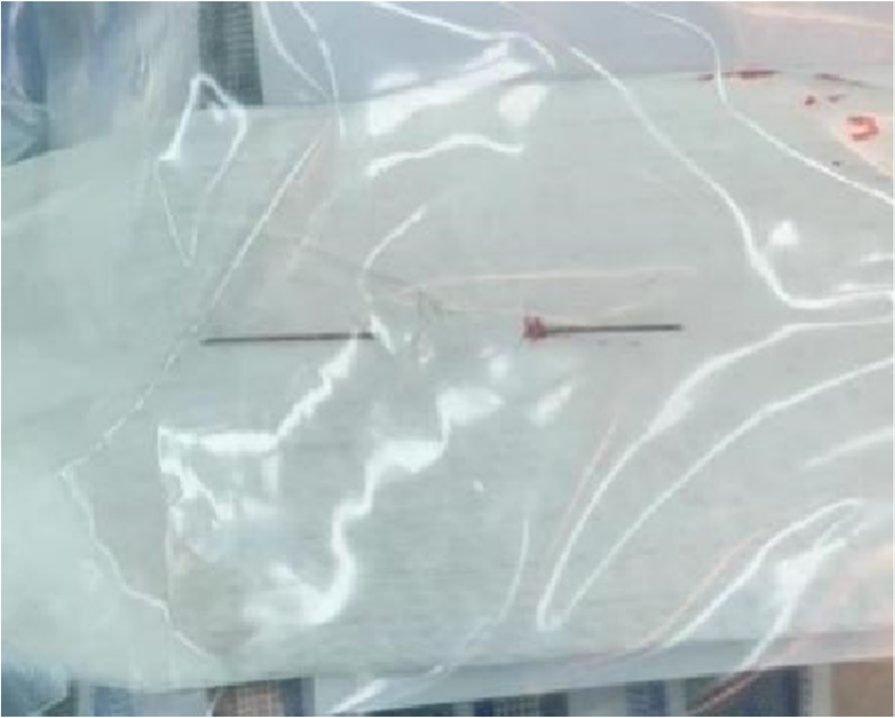
August 30, 2023, the second needle extracted from the patient’s right buttock

On the 45th day following surgery, the patient presented with a noticeable bulge and pronounced tenderness in the localized area of the right buttock. Physical examination revealed a localized skin bulge, situated approximately 5 cm above the surgical scar on the right buttock, accompanied by tenderness and a sensation of a foreign object upon palpation (Fig. 9). An emergent local anesthetic examination was conducted. Intraoperative X-ray found that the residual needle was located within the right buttock, perpendicular to the surface of the body, and approximately 1.5 cm away from the skin. Subsequently, the needle was completely extracted through a local incision (Fig. 10) and the patient experienced a complete resolution of pain the day after the procedure. During the follow-up, the patient noted a slight decrease in the length and rigidity of the penis during erections compared to the preoperative state.

### Literature review

We searched the PubMed and Embase databases for similar cases and compared the findings across all the cases. 15 articles were finally included and 17 cases were extracted. All included cases were self-insertion of needle-like foreign bodies into the cavernous body of the penis (see Table 1).

**Table 1:** Comprehensive comparison among all reported cases of retained penile needles

first author	No. of patients	age	cause	Palpable needle on PE	Interval of needle retrieval	Imaging modality	needle location	Remove location	Local vs. general anesthesia
this case	1	47	Self implantation	NO	22 h	CT	pelvic cavity	pygal	General anesthesia
Mark (1991) [1]	1	67	ICI	YES	Immediate	X-ray	NA	Local area of the penis	Local anesthesia
Beer (1992) [2]	1	61	ICI	NO	2 d	X-ray. Ultrasound failed to localize the needle	Intracorporal. At the base of the left corpus cavernosum	Local area of the penis	Local anesthesia (penile block)
Nazli (1993) [3]	1	58	ICI	YES	Immediate	X-ray	Intracorporal. Left corpus cavernosum	Local area of the penis	Local anesthesia
Greenstein(1997) [4]	2 case 1	69	ICI	NO	6 wk	X-ray to confirm needle presence on initial presentation	NA	Local area of the penis	Needle was palpable at time of retrieval. No anesthesia used at all.
	case 2	81	ICI	NO	4 d	X-ray to confirm needle presence on initial presentation	NA	Local area of the penis	Needle was palpable at time of retrieval. No anesthesia used at all.
Iacono (1998) [5]	1	50	ICI	NO	Immediate	Ultrasound	Intracorporal. Distal third of the right corpus cavernosum	Local area of the penis	No anesthesia used at all
Bandi (2005) [6]	1	44	ICI	N0	2 wk, nonoperative management	X-ray	NA	Local area of the penis	No
Shamloul(2005) [7]	1	32	ICI	N0	Immediate	X-ray	Intracorporal. Midshaft of left corpus cavernosum	Local area of the penis	Local anesthesia (penile block)
Hsiao (2013) [8]	1	42	ICI	No, only superficial palpation performed due to fear of needle stick	Immediate	X-ray	Intracorporal. Midshaft of right corpus cavernosum	Local area of the penis	General anesthesia
Banerji(2016) [9]	1	62	ICI	YES	Immediate	X-ray	Under Buck’s fascia (right midshaft)	Local area of the penis	General anesthesia
Wren (2018) [10]	1	55	ICI	NO	3 d	X-ray	Intracorporal. Left corpus cavernosum	Local area of the penis	General anesthesia, penile degloving
Pan (2019) [11]	1	51	ICI	NO	Several months	Ultrasound and CT scan	Under Dartos fascia (right side of penile base)	Local area of the penis	General anesthesia
Collaço(2020) [12]	1	54	ICI	NO	Immediate	X-ray and ultrasound	Intracorporal. Left corpus cavernosum	Local area of the penis	General anesthesia
Lazaraviciute(2021) [13]	1	82	ICI	NO	1 d	X-ray and ultrasound	Intracorporal. Left corpus cavernosum	Local area of the penis	General anesthesia
Mai (2021) [14]	1	64	ICI	YES	Immediate	X-ray and ultrasound	Under Dartos fascia (left midshaft)	Local area of the penis	General anesthesia
Kirolos(2023) [15]	1	70	ICI	NO	2 d	Ultrasound	Intracorporal. Midshaft of right corpus cavernosum	Local area of the penis	General anesthesia

*ICI* intracavernosal injection therapy

## Discussion

Penile foreign bodies, such as those in the foreskin, are relatively common [16]. A small number of individuals usually surgically implant beads of various materials and shapes beneath the foreskin through a dorsal foreskin incision, as elucidated in existing literature [16]. One optional remedy for ED is ICI. Since 1982, some ED sufferers have been consistently self-administering injections of medications, such as botulinum toxin, directly into the corpus cavernosum as a means of treatment. However, this self-injection method poses significant challenges, and the use of ICI in China remains relatively limited due to the requirement for specialized expertise and associated discomfort [17]. Through the literature review, there have been 16 reported cases of self-insertion of needle-like foreign bodies into the cavernous body of the penis. And they were all ICI-associated needle breakage and retention abroad, with the needle being inserted into the penile body rather than the glans at the time of injection. And the longer the delay, the easier it is for the needle to move its position. Finally, the needles all be removed through a local incision in the penis. As of yet, no similar case has been reported in China [15]. In particular, this case encompasses the simultaneous presence of two uncommon clinical conditions: foreign bodies within the penile cavernous body, and the coexistence of glans cleft and hypospadias in the patient.

Ultrasound, X-ray, and CT are viable diagnostic options for the detection and localization of foreign bodies within the penile cavernous body. 3D reconstruction of CT proves highly beneficial in precisely locating deeply embedded foreign tissues, enabling surgeons to devise effective surgical plans aimed at protecting the cavernous body tissues and minimizing the risk of postoperative ED [18].

Specifically, in cases involving parenchymal organs, the retention and removal of needle-like foreign entities can be very challenging and present surgeons with “needle in a haystack” situation. Preoperative 3D reconstruction of CT and intraoperative X-ray can be instrumental in precisely locating the needles. The unpredictable displacement trajectory of the foreign body represents the primary challenge. In this case, the complete removal of the needles from the patient required over a month as they migrated proximally within the cavernous body before penetrating the subcutaneous tissue of the sacrococcygeal region. It is thought that variations in intra-abdominal pressure, penile tissue deformation, hemodynamics, and muscular activity collectively contribute to the movement of the needles. In case the foreign body has migrated deeper into the tissues as a result of muscle movement, and the patient does not exhibit any specific symptoms or risks of macrovascular injury-related bleeding, close surveillance of its movement can be implemented. Surgical intervention should only be initiated once the foreign body has reached a suitable position to prevent excessive tissue damage and functional impairment. The second difficulty pertains to the challenge of the control of hemorrhage and the prevention of substantial harm to erectile tissue during the incision of the penile cavernosa for needle extraction. The third dilemma is related to the spongy tissue structure of penis, which may reduce the surgeon’s tactile feedback and increase the likelihood of missing thin needles even with intraoperative fluoroscopic guidance. Therefore, to prevent excessive manipulation that could potentially push the needle further into deeper penile tissues and consequently complicate its retrieval, it is recommended that emergency departments seek assistance from experienced andrologists first in cases of intracorporeal needle break in the penile cavernous body [15].

When the anatomical structure of the penis is normal, inserting a needle from the glans to the corpus cavernosum can result in excruciating agony. The patient in this case presented with a penile deformity and experienced only mild discomfort during needle insertion. It remains unclear whether the presence of this deformity is correlates with decreased penile nociception [18]. It is known that individuals with mental health issues may occasionally engage in deviant sexual activity, such as inserting foreign objects into the urethra [19]. The reasons for the insertion of foreign bodies into the genitourinary tract could be sexual gratification, psychiatric, accidental, curiosity, especially among children, or therapeutic [20]. The individual involved in this case, despite engaging in a behavior that may appear peculiar to the majority, is obviously a normal, mature member of society, without any known psychiatric abnormalities. However, it is only speculative at present regarding the psychological issues underlying the patient’s abnormal behavior, as the patient refused any discussions pertaining to this matter. Nevertheless, when dealing with such unconventional circumstances, it is essential for clinicians to be mindful of the potential psychological issues that the patient may be experiencing. Communication approaches should be carefully considered when interacting with the patient and their family. Additionally, it is suggested that a psychiatric evaluation should be recommended to discover any underlying mental health disorders, thus reducing the risk of recurrence [21] .

## Conclusion

This article presents a unique case involving the self-insertion of needles into the cavernous body of the penis, which were eventually removed in a staged procedure from the buttocks. To our knowledge, this is the first case of migrating foreign bodies within penis in China. In such cases, it is of utmost importance to engage experienced andrologists to prevent excessive manipulation and further needle penetration into the penile tissue. In case the foreign body has migrated deeper into the tissues and the patient does not exhibit any specific symptoms or risks of macrovascular injury-related bleeding, close surveillance of its movement can be implemented. Surgical intervention can be initiated once the foreign body has reached a suitable position to prevent excessive tissue damage and functional impairment. Moreover, a psychiatric evaluation should be recommended for patient to discover any underlying mental health disorders.

## Data Availability

No dataset was generated or analyzed during this study.

## Abbreviations

CT: Computed Tomography
ED: Erectile dysfunction
ICI: Intracavernosal injection therapy
